# Exchanging knowledge across international borders: building a new network for the RCGP’s global members

**DOI:** 10.3399/bjgpopen17X101313

**Published:** 2017-11-15

**Authors:** Sandra Mather, Terry Kemple, Asha Abdillahi

**Affiliations:** 1 Head of International, Royal College of General Practitioners, London, UK; 2 President, Royal College of General Practitioners, London, UK; 3 International Project Development Manager, Royal College of General Practitioners, London, UK

**Keywords:** International and Overseas Network, Global members, General Practice, Professional networks, Royal College of General Practitioners

The Royal College of General Practitioners (RCGP) has a growing and diverse global membership of 3048 members (Box 1) based across 86 countries outside the UK. This represents approximately 6% of the total membership of the RCGP. In March 2017, it launched a new International and Overseas Network (ION) to link its global members with 'knowledge, support and inspiration' in their professional practice,^1^ and to improve its professional global connections.

Networks can be described as 'complex adaptive systems' with multiple non-linear interactions between members of the system.^2–3^ Network structures vary along a continuum. At one end is a network defined as a diffuse decentralised structure comprising individuals (sometimes called communities of practice) or informal alliances of organisations. At the other end is a hierarchical network organisation with a centralised hub and spoke structure and clear governance systems comprising either organisations or individuals. ^3–5^

**Box 1.** Definitions of international and overseas member of the RCGP

International member (IM)	A GP who has passed an RCGP international membership exam at any one of nine accredited exam sites and has chosen to apply for membership. They are classified as international members and use the post-nominals MRCGP[INT].
Overseas member (OM)	A GP who has passed the RCGP membership exam in the UK and has chosen to apply for membership, which they retain after leaving the UK to work in another country. They are classified as overseas members and can use the post nominals MRCGP.

## The RCGP as a network

When the RCGP was founded in 1952, it began as an informal network but was developed into the formal institutionalised network that is now the largest medical royal college in the UK. This history helps in understanding the development of the ION, the profession, and similar associations in the countries where the RCGP's global members are working.

All professional networks function at two levels: formal and informal. The formal network is built around the membership body itself with systems, processes, and structures to adhere to, whereas the informal networks are clustered around smaller, closely linked groups of individual professionals. Millar and Choi^6^ argue that formal networks are essential to transfer explicit knowledge but that informal 'sub-networks' are crucial for tacit knowledge transfer and, therefore, holistic knowledge exchange. These informal sub-networks are built around social norms and trust between members and are sometimes defined by geographical boundaries, personal interests, and connections between members. The relationships and strength of the links that people develop in the early stages of the formation of a network form the basis of how it is likely to behave in future. These links bind the network together and maintain it as it develops because, first and foremost, 'members are the heart and soul of network organisations'.^7^ Granovetter describes a network tie’s strength as being a mixture of 'the amount of time, the emotional intensity, the intimacy (mutual confiding), and the reciprocal services which characterise the tie'.^8^

### Principles for establishing the ION

Bennett^5^ set out 10 principles for network success (Box 2) and these were applied in the establishment of the ION.

**Box 2.** Bennett’s 10 principles for network success

The importance of time Ownership, trust, and confidence Distributed leadership Transparent, accountable, shared decision making Basis of unity: common purpose and goal Shared values and norms of behaviour Understanding each other, sharing skills, and approaches Ad hoc relations between members ‘Quick wins’: the value of shared successes and individual benefits ‘Unity with diversity’: difference, inclusion, and boundaries

### Recruiting the 14 Founder Members of the ION

The RCGP established this new global network with a core group of 14 GPs as Founder Members (FMs). These members were recruited from the 16 countries where the RCGP has strategic partnerships and/or the highest numbers of members. All 2154 members in these 16 countries were emailed with an invitation to apply, and 124 applications were received (response rate 5.8%).

#### Criteria for selection

All applicants were asked to give three reasons for their application as FM of ION, and the name and membership number of a peer in support of their application.

#### Recruitment process

Applications were considered by two reviewers, who selected those who demonstrated the greatest evidence of networking experience and the potential to actively build networks in their region.

### Successes in the first 6 months

#### Applying Bennett’s 10 principles for network success

Bennett’s 10 principles were crucial in establishing this new network and were discussed with the FMs at the launch event in March 2017. All 10 principles contributed to varying extents to the way the FMs interacted on the launch day, how they developed the action plan, and how they interact as they implement this plan in the first 6 months of the network’s life. The distributed leadership model has worked particularly well (principle 3) and was quickly implemented, with six of the FMs volunteering to lead on aspects of the action plan. A 'quick win' (principle 9) was the RCGP’s decision to respond immediately to the network’s request to allocate a manager to support the network.

#### First dedicated survey to international and overseas members

The FMs of the network successfully contributed to the development of the first dedicated survey to all international and overseas members. This was issued to 3048 members between June and July 2017. The 18.1% response rate was higher than expected, based on other similar research in membership organisations. The survey uncovered two key messages from members:

- International members want the RCGP to advocate on their behalf to have their MRCGP[INT] qualification recognised internationally.
- Overseas members want the RCGP to advocate on their behalf to have their MRCGP qualification recognised in other countries, and also to simplify the revalidation process so that those who wish to can return to work in the UK.

### Challenges in the first 6 months

#### Arranging meetings with 14 members working in different time zones

This has been a challenge to organise. To avoid meetings falling too early or too late in the clinical day for some members, two duplicate meetings were held on the same day: one in the morning and one in the afternoon, both with the same agenda.

#### Digital communication

Low bandwidth in some countries where members work has meant that some digital tools to connect visually, or via an online knowledge exchange digital platform, have not been used as much as was expected at the outset of the network. Other digital tools have been tried, but the lowest common denominator, and the communication tool that all members in all countries can always access, is email. So, communication has been primarily via email and meetings have been held via toll-free telephone calls where other digital technology has not been usable.

#### Globalisation of membership bodies

Many membership organisations are increasing their global reach.^9–11^ However with global expansion comes challenges, including how to bring global members together (virtually or in person, across international borders), and to what extent they should be allowed to evolve organically or be provided with governance structures and central secretariat support. Networks are looking to digital solutions to support their expansion and support their global membership. Barkan^11^ and Hanson^9^ both challenge membership organisations, firstly, to ask themselves whether they are truly global organisations or whether they are merely national organisations with a few international members; and, secondly, if they are global, to provide a positive membership experience for all members.

## Summary

The RCGP is a global membership organisation that exists to improve the standards of care for patients. It exists to help its members, and their standards of general practice. The RCGP embraces the diversity, the experience, and the talents of its global membership to enable it, everywhere, to strengthen its role as an effective global organisation. This increases its voice and influence internationally, enabling it to continue to maintain and develop the highest standards of general medical practice globally.

The RCGP is an established large, hierarchical, and governance-driven global professional membership body and, in March 2017, it invited its global members to set up a new ION. To spread best practice faster, one must find oneself a better network. This new ION represents the RCGP — a large, formal professional network — growing a new, smaller, and less formal global network to find and spread best practice faster.

## References

[1] Royal College of General Practitioners (2017). International and Overseas Network. http://www.rcgp.org.uk/rcgp-near-you/rcgp-international/international-and-overseas-network.aspx.

[2] Sweeney K, Griffiths F (2002). Complexity and healthcare: an introduction.

[3] Taschereau S, Bolger J (2006). Networks and capacity: a theme paper prepared for the study “Capacity, Change and Performance*”*. http://portals.wi.wur.nl/files/docs/SPICAD/13.%20Networks%20and%20capacity%20(ECDPM).pdf.

[4] Ramalingam B (2011). Mind the network gaps. https://www.odi.org/sites/odi.org.uk/files/odi-assets/publications-opinion-files/7110.pdf.

[5] Bennett R (2014). Establishing a national coordinating body for non-profit organisations. Research report Praxis Paper 29. https://www.intrac.org/resources/praxis-paper-29-establishing-national-coordinating-body-non-profit-organisations/.

[6] Millar CCJM, Choi CJ (2009). Networks, social norms and knowledge sub-networks. J Bus Ethics.

[7] Sluijs-Doyle J (2017). Capacity building in network organisations.

[8] Granovetter MS (1973). The Strength of Weak Ties. Am J Sociol.

[9] Hanson W (2013). Going global: growing your international membership.

[10] PARN (2013). The Network Response on Member Networks.

[11] Barkan T (2017). Global growth strategies for the international association. Your guide to developing a global association strategy. http://static1.1.sqspcdn.com/static/f/726646/27505294/1490719955660/Global+Growth+Strategies+-+2017.pdf?token=vu8C3J%2BgtfkoTLipKw7kowwEqpA%3D.

[12] Levin DZ, Walter J, Murnighan JK (2011). Dormant ties: the value of reconnecting. Organ Sci.

